# Does invasive acupuncture improve postoperative ileus after colorectal cancer surgery? A systematic review and meta-analysis

**DOI:** 10.3389/fmed.2023.1201769

**Published:** 2023-08-25

**Authors:** Xiaohu Zhao, Shangkun Si, Xin Liu, Jingxuan Liu, Dongbin Zhang, Yuejun Mu, Aihua Hou

**Affiliations:** ^1^College of Traditional Chinese Medicine, Shandong University of Traditional Chinese Medicine, Jinan, China; ^2^Department of Oncology, Yantai Hospital of Traditional Chinese Medicine, Yantai, China; ^3^Department of Anesthesiology, Affiliated Hospital of Shandong University of Traditional Chinese Medicine, Jinan, China

**Keywords:** acupuncture therapy, meta-anlaysis, postoperative complications, surgical oncology, Traditional Chinese Medicine

## Abstract

**Background:**

Postoperative ileus (POI) is one of the main complications after colorectal cancer (CRC) surgery, and there is still a lack of effective treatment. At present, the evidence for improvement of POI by invasive acupuncture (manual acupuncture and electroacupuncture, IA) is limited. This meta-analysis of randomized controlled trials (RCTs) aims to systematically review and evaluate the effect of IA in improving POI after CRC surgery.

**Methods:**

This meta-analysis was reported according to PRISMA statement and AMSTAR guidelines. The retrieval time was from the inception to February 2023. The RCTs were screened by searching the databases (PubMed, Ovid, Embase, Cochrane Library, China National Knowledge Infrastructure, VIP Database, Sinomed Database, and WANFANG). Two independent investigators screened and extracted the data, assessed the risk of bias, and performed statistical analysis. The statistical analysis was carried out by RevMan5.3. The PROSPERO International Prospective Register of Systematic Reviews received this research for registration (CRD42023387700).

**Results:**

Thirteen studies with 795 patients were included. In the primary outcome indicators: the IA group had shorter time to the first flauts [stand mean difference (SMD), −0.57; 95% CI, −0.73 to −0.41, *p* < 0.00001], shorter time to the first defecation [mean difference (MD), −4.92 h, 95% CI −8.10 to −1.74 h, *p* = 0.002] than the blank/sham stimulation (B/S) group. In the secondary outcome indicators: the IA group had shorter time to the first bowel motion (MD, −6.62 h, 95% CI −8.73 to −4.50 h, *p* < 0.00001), shorter length of hospital (SMD, −0.40, 95% CI −0.60 to −0.21, *p* < 0.0001) than the B/S group. In terms of the subgroup analysis: IA associated with enhanced recovery after surgery (ERAS) group had shorter time to the first flauts (MD, −6.41 h, 95% CI −9.34 to −3.49 h, *p* < 0.0001), shorter time to the first defacation (MD, −6.02 h, 95% CI −9.28 to −2.77 h, *p* = 0.0003) than ERAS group.

**Conclusion:**

Invasive acupuncture (IA) after CRC surgery, acupuncture or electricacupuncture with a fixed number of times and duration at therapeutic acupoints, can promote the recovery of POI. IA combined with ERAS is better than simple ERAS in improving POI.

**Systematic review registration:**

https://www.crd.york.ac.uk/PROSPERO/display_record.php?RecordID=387700, identifier CRD42023387700.

## 1. Introduction

Postoperative ileus (POI) is one of the main postoperative complications of colorectal cancer (CRC) surgery, and recent research shows that its incidence rate is 13.5% (1). The recovery of POI usually takes 4 days, and the main clinical manifestations are delayed exhaust and defecation, abnormal bowel sounds, abdominal distention, nausea, and vomiting (2, 3). In addition, POI also extended the time and expenses for hospitalization (4). At present, basic treatments, such as fasting, nutritional support, maintaining water, electrolyte, and acid–base balance, are used to treat POI. Other therapies like drugs to promote gastrointestinal motility, chewing gum, and so on, are also used (5). However, the clinical efficacy of the existing treatment schemes is limited. Therefore, finding a new treatment to prevent POI has become an important issue so that patients with colorectal cancer can recover quickly during postoperative period (6).

Acupuncture is a non-drug, safe and inexpensive treatment. Some existing clinical studies show that acupuncture and related therapies can effectively improve the POI in surgical operations (7). However, external treatment were combined with invasive acupuncture (IA) in the intervention measures, such as moxibustion, acupoint application, and transcutaneous acupoint electrical stimulation (8). Therefore, we want to explore whether IA alone can improve POI after CRC surgery. And we include related randomized controlled trials (RCTs) for this meta-analysis so as to clarify its effect.

## 2. Materials and methods

### 2.1. Protocol and registration

The Preferred Reporting Items for Systematic Reviews and Meta-Analyses (PRISMA) standards were followed while reporting this study. Supplementary Table S2 contains the PRISMA checklist. This study was also followed the guidelines of the Cochrane Handbook for Systematic Reviews of Interventions. PROSPERO International prospective register of systematic reviews was where the review procedure was registered (CRD42023387700).

### 2.2. Search strategy

The following databases were searched for this meta-analysis: PubMed, Ovid, Embase, Cochrane Library, China National Knowledge Infrastructure, VIP Database, Sinomed Database, and WANFANG Medical. It was reported in accordance with the PRISMA declaration and AMSTAR criteria. The search period spanned from beginning until February 2023. High-quality RCTs were gathered by two independent researchers. According to the search’s approach, full-text search was carried out (Supplementary Table S3).

### 2.3. Eligibility criteria

Inclusion criteria include (1) Research type: RCTs on the preventive and therapeutic effects of IA (manual acupuncture and electroacupuncture) on POI after CRC surgery; Language is not limited. (2) Research objects: Patients with CRC undergoing IA during perioperative period; Age, gender and nationality are not limited. (3) Intervention measures: The acupuncture group received manual acupuncture (MA) or electroacupuncture (EA) during perioperative period; The blank/sham stimulation group(B/S) did not receive any treatment or stimulated non-meridian points.

Exclusion criteria include (1) Non-invasive acupuncture acupoint stimulation therapy, such as transcutaneous acupoint electrical stimulation, acupoint application, auricular point pressing beans, etc. (2) Intervention measures are IA combined with other Chinese medicine treatments, such as oral Chinese medicine decoction. (3) Non-colorectal cancer surgery patients or other intervention methods entered the research literature. (4) Literature with incomplete original text or ending index cannot be obtained. (5) Non-RCT, systematic review or comments, editorials, letters, meetings, and animal trials.

### 2.4. Outcome indicator

According to the effect of IA on preventing and treating POI, the main outcome indicators are: (1) Time to the first flauts; (2) Time to the first defecation; Secondary outcome indicators: (3) Time to the first bowel motion; and (4) Length of hospital.

### 2.5. Data extraction and quality assessment

According to the eligibility criteria above, two independent investigators preliminary screened through the title and abstract, and then the full text. The author’s name, publishing years, size of the sample, intervention methods, outcome indicators, and other data were extracted from the final screened literature. In case of disagreement, any potential disagreement shall be submitted to the correspondent for arbitration.

According to the Cochrane systematic review handbook 5.1 and its suggested risk of bias assessment technique, the caliber of the included studies was assessed. Random sequence generation, allocation concealment, blinding of trial participants and staff, blinding of outcome assessors, inadequate outcome data, selective reporting, and other biases were all examined in the research. The bias assessment’s findings were “low risk,” “high risk,” and “unclear.” Two researchers independently completed the quality evaluation. Conflicts were resolved through mediation by the corresponding author.

### 2.6. Statistical analysis

Utilizing the program RevMan 5.3, statistical data analysis was carried out. The Standard mean difference (SMD) or Mean difference (MD) and its 95% CI are statistically described and the effect quantity is combined. Inter-study heterogeneity was assessed using chi-square test with a test level of *α* = 0.1, and the degree of heterogeneity was observed based on *I^2^* values (9). The studies with clinical and methodological homogeneity were combined, and if *p* ≥ 0.1 and *I^2^* ≤ 50%, the included studies had good homogeneity and were analyzed using a fixed-effects model for meta-analysis; if *p* < 0.1 and *I^2^* > 50%, it was considered that there was significant heterogeneity among the included study literature, and subgroup analysis treatment or sensitivity analysis was needed to find the source of heterogeneity; if there was no significant clinical heterogeneity, the random-effects model was selected for merging; sensitivity analysis was performed when there were large weight items to check the stability of the results; if the heterogeneity was large, meta-analysis was not performed, and only descriptive analysis was performed. If the number of literatures was sufficient, funnel plots were used to determine whether there was publication bias (10).

## 3. Results

The GRADE evidence profiles and summary of findings table was shown in Table 1.

**Table 1:** GRADE evidence profiles and summary of findings table.

Outcome (studies)	No. of participants		Risk of bias	Inconsistency	Indirectness	Imprecision	Other considerations	Overall certainty of evidence	Anticipated absolute effects (95% CI)
	IA	B/S							
Time to first flauts (13 RCTs)	316	316	Serious	Not serious	Not serious	Not serious	None	⨁⨁⨁◯ Moderate	SMD −0.57 lower (−0.73 lower to −0.41 lower)
Time to first defecation (hours; eight RCTs)	189	191	Serious	Not serious	Not serious	Not serious	None	⨁⨁⨁⨁ High	MD −4.92 lower (−8.10 lower to −1.74 lower)
Time to first bowel motion (hours; nine RCTs)	200	205	Very serious	Not serious	Not serious	Not serious	None	⨁⨁◯◯ Low	MD −6.62 lower (−8.73 lower to −4.50 lower)
Length to hospital (seven RCTs)	214	215	Not serious	Not serious	Not serious	Not serious	None	⨁⨁⨁⨁ High	SMD −0.40 lower (−0.60 lower to −0.21 lower)
ERAS subgroup time to first flauts (hours; four RCTs)	141	142	Serious	Not serious	Not serious	Not serious	None	⨁⨁⨁◯ Moderate	MD −6.41 lower (−9.34 lower to −3.49 lower)
ERAS subgroup time to first defecation (hours; four RCTs)	141	142	Serious	Not serious	Not serious	Not serious	None	⨁⨁⨁◯ Moderate	MD −6.02 lower (−9.28 lower to −2.77 lower)

IA, invasive acupuncture; B/S, the blank/sham stimulation; SMD, stand mean difference; and MD, mean difference.

### 3.1. Literature search

**Figure 1:**
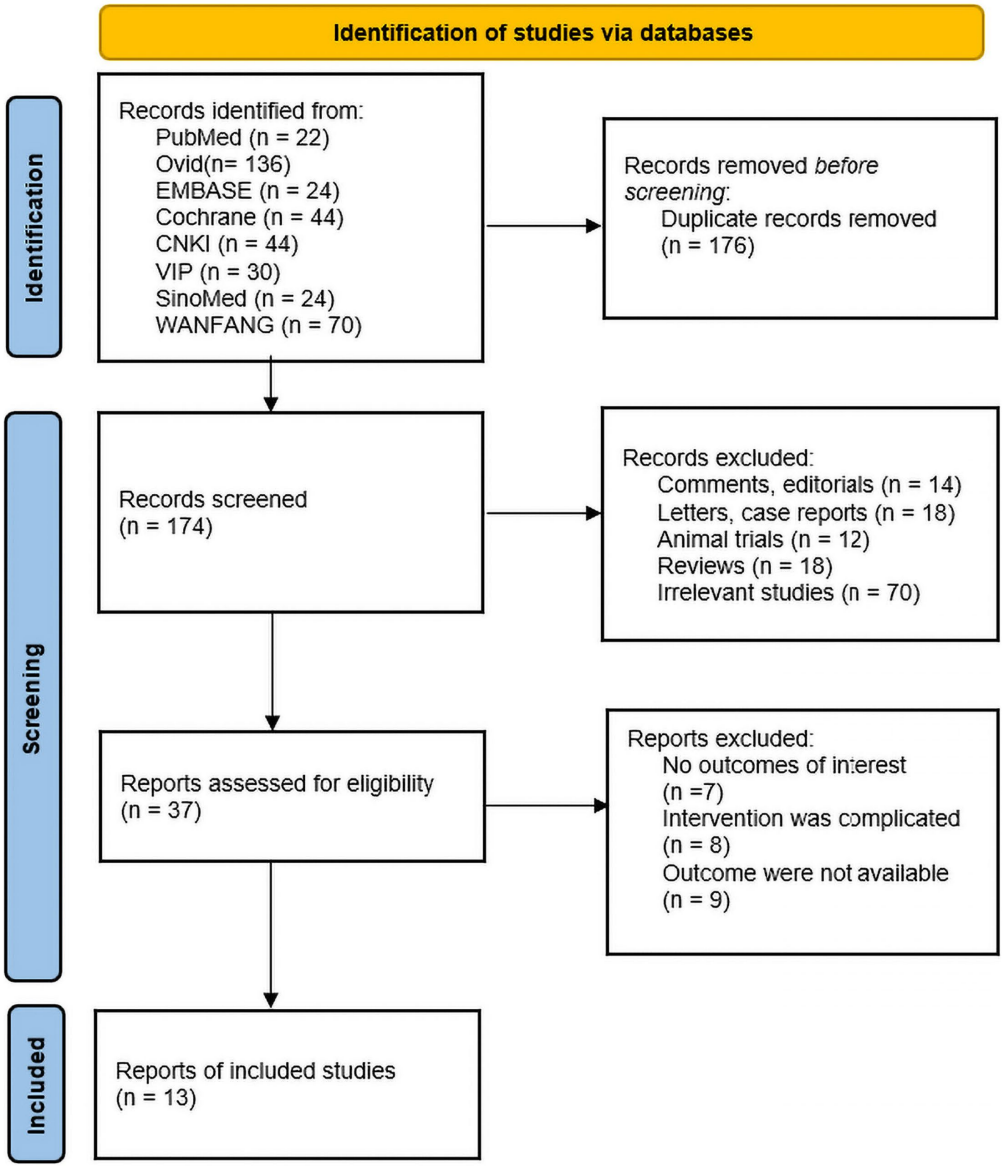
Preferred Reporting Items for Systematic Reviews and Meta-Analyses (PRISMA) flow diagram.

A total of 350 relevant literatures were obtained from the search, 176 duplicates were excluded, 137 were eliminated based on title and abstract, and then 24 were excluded based on full text, resulting in the inclusion of 13 studies that met the study requirements, all in China, including one conducted in Hong Kong. There were 395 patients in the IA group and 400 patients in the B/S group, for a total of 795 patients. There of the included studies explicitly mentioned the use of open surgery, four explicitly mentioned the use of laparoscopic surgery, and the other studies described only part of the type of surgery; four studies used enhanced recovery after surgery (ERAS); nine studies used EA, four studies used MA; one study performed acupuncture 1 day before surgery; and the remaining studies were performed postoperatively. The literature screening process was shown in Figure 1. The basic characteristics of the included studies were shown in Tables 2, 3.

**Table 2:** Study characteristics.

Authors, year	Surgery	Cancer type	Intervention	Patients	Sample size		Outcome
					T	C	
Yang et al. (11)	Laparoscopic surgery	CRC	EA	Adult	35	35	①②④
Wang et al. (12)	Laparoscopic radical surgery	CRC	MA	Adult	30	30	①②③④
Liu et al. (13)	Laparoscopic surgery	CRC	MA	Adult	33	35	①②③④
Cai et al. (14)	Radical surgery	CRC	MA	The aged	32	31	①②
Wang et al. (15)	Usual surgery	RC	EA	Adult	20	20	①②③
Li et al. (16)	Radical surgery	CRC	EA	Adult	42	40	①②③④
Mai et al. (17)	Open surgery	CRC	EA	Adult	20	20	①②③
Xiao et al. (18)	Radical surgery	CC	MA	The aged	30	30	①②④
Si et al. (19)	Radical surgery	CC	EA	Adult	25	25	①③
Zhang et al. (20)	Open surgery	CRC	EA	Adult	19	20	①②③④
Ng et al. (21)	Laparoscopic surgery	CRC	EA	Adult	55	55	①③④
Meng et al. (22)	Open surgery	CC	EA	Adult	35	40	①③
Niu et al. (23)	Radical surgery	CC	EA	Adult	19	19	①

CRC, colorectal cancer; RC, rectal cancer; CC, colon cancer; MA, manual acupuncture; and EA, electroacupuncture; ①: Time to first flatus; ②: Time to first defaecation; ③: Time to first bowel motion; and ④: Length of postperative hospital stay.

**Table 3:** Details of POI interventions.

Author, years	EA or MA procedures	Control interventions	Acupoints
Yang et al. (11)	Postoperative day 2–6/time of discharge; 30 min, once a day; United ERAS	ERAS	Bil (ST36, ST25)
Wang et al. (12)	Postoperative day 1–5; 30 min, once a day	Routine treatment	Bil (ST36, ST25, and PC6); RN6, RN10, RN12, and RN13
Liu et al. (13)	Postoperative day 1–4; 30 min, once a day; United ERAS	ERAS	Bil (ST36, ST37, LI4, and PC6)
Cai et al. (14)	Attached within 2 h after surgery and lasted for 3 days, pressure every 8 h; United ERAS	ERAS	Uk (TF4, CO4, CO6, CO7, CO17, ST36, ST37, ST25, and PC6)
Wang et al. (15)	Postoperative day 1–10; 20 min, once a day	Routine treatment	Bil (LI9, ST39, and ST25); RN12
Li et al. (16)	Once postoperative 6 h, postoperative day 1 to time of ending ileus; 30 min, twice a day; United ERAS	ERAS	Uk (ST36, PC6)
Mai et al. (17)	Preoperative 30 min/1 day; 30 min, once	Routine treatment	R (ST36, ST37, ST39, ST25, and PC6); RN12
Xiao et al. (18)	Postoperative day 1–14; 30 min, twice a day	Routine treatment	Bil (LU5, LU7, LI4, SJ6, ST36, and SP6)
Si et al. (19)	Postoperative 24 h to time of ending ileus; 20 min, once a day	Routine treatment	Bil (ST36, ST37, ST25, SP6, and LI4)
Zhang et al. (20)	Once postoperative 30 min, postoperative day 1–4; 30 min, once a day	Sham acupuncture	Bil (ST36)
Ng et al. (21)	Postoperative day 1–4/time of ending ileus; 20 min, once a day	Sham acupuncture	Uk (ST36, SP6, LI4, and SJ6)
Meng et al. (22)	Postoperative day 1–6/time of ending ileus; 20 min, once a day	Routine treatment	Uk (SJ6, GB34, ST36, and ST37)
Niu et al. (23)	From postoperative day 1, 15 min, twice a day	Routine treatment	Uk (ST36, ST37, and PC6)

Bil, bilateral; R, right; Uk (unknown), it was not clear whether it was unilateral or bilateral; ERAS, enhanced recovery after surgery; ST25,Tianshu acupoint, ST36, Zusanli acupoint; ST37, Shangjuxu acupoint; ST39, Xiajuxu acupoint; RN6, Qihai acupoint; RN10, Xiawan acupoint; RN12, Zhongwan acupoint; RN13, Shangwan acupoint; LI4, Hegu acupoint; LI9, Shanglian acupoint; LU5, Chize acupoint; LU7, Lieque acupoint; SJ6, Zhigou acupoint; SP6, Sanyinjiao acupoint; GB34, Yanglingquan acupoint; PC6, and Neiguan acupoint. Ear points: TF4 Shenmen, CO4 Wei, CO6 Xiaochang, CO7 Dachang, and CO17 Sanjiao.

### 3.2. Quality assessment of included studies

(1) Random sequence generation: four papers used random number table method; two papers used computer software to generate random groupings; two papers used random zone grouping method; one paper used closed envelopes; two papers only mentioned random and did not describe the method of random sequence generation; one paper mentioned minimization; one paper did not mention random. (2) Allocation concealment: three papers used closed envelope allocation concealment; two papers mentioned allocation concealment without specific methods, and the rest did not mention allocation concealment. (3) Blinding of subjects and trial personnel: five papers mentioned blinding of subjects and investigators; one mentioned using single blinding, and the rest did not mention blinding. (4) Blinding of outcome assessors: five papers mentioned blinding of outcome index assessors, and the rest did not describe it. (5) Incomplete outcome data: one study mentioned the reason why some of the outcome indicators did not appear, but the proportion of missing outcome indicators was not enough to have a significant impact, and the rest of the studies had no missing data. (6) Selective reporting: three studies had access to the study protocol and eventually reported the expected outcome indicators, while the rest had incomplete information and it was difficult to determine whether there was a risk of selective reporting of outcomes. (7) Other bias: there was no evidence or information to determine whether there was other serious risk of bias in the included studies.

**Figure 2:**
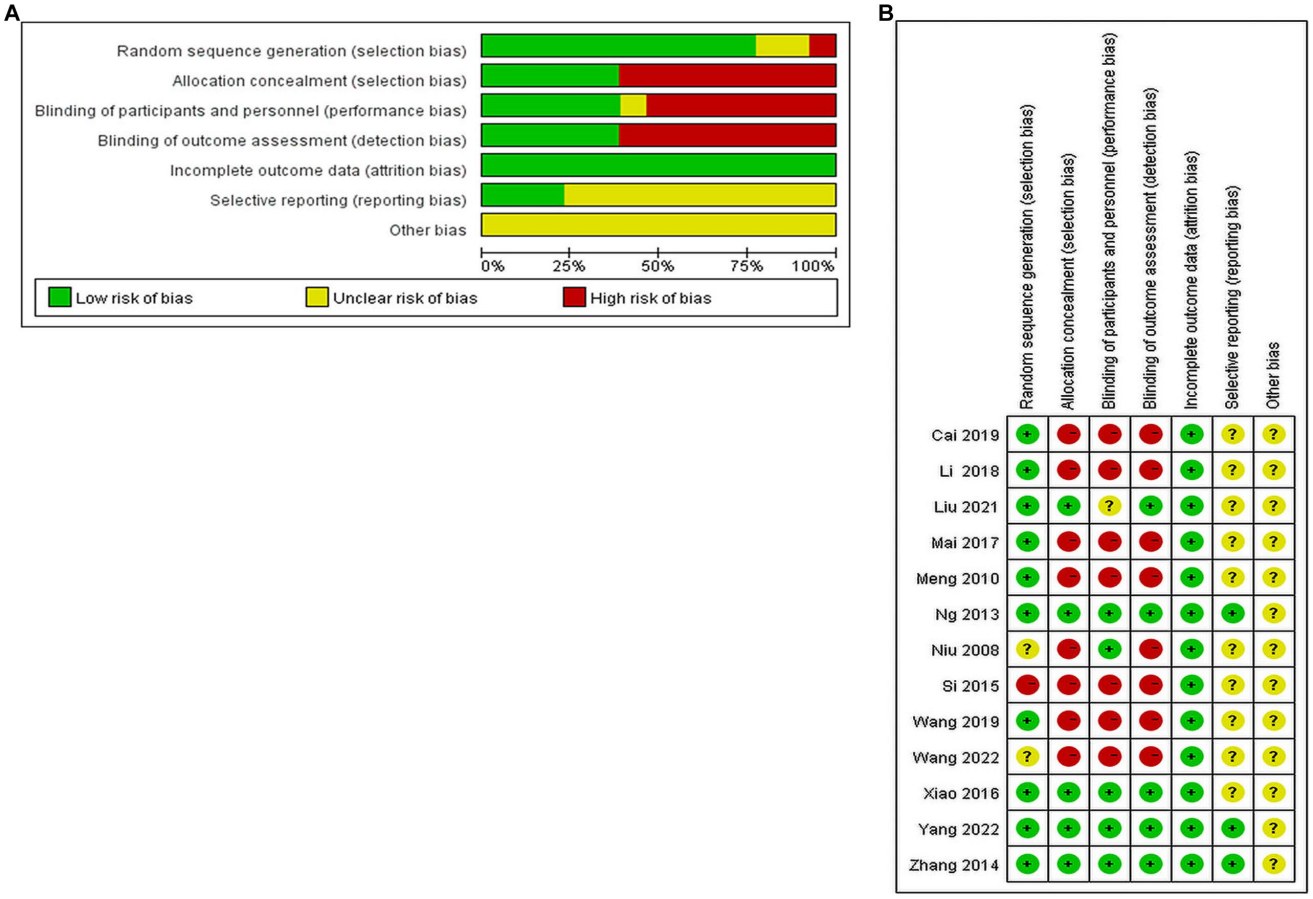
Percentage plot of the risk of bias of the included studies. **(A)** Risk of bias graph. **(B)** Risk of bias summary.

Of the 13 included publications, our quality assessment using the Cochrane Risk of Bias Assessment Tool showed that the overall quality of the total literature was moderate (Figure 2).

### 3.3. Primary outcome indicators

#### 3.3.1. Time to the first flauts

**Figure 3:**
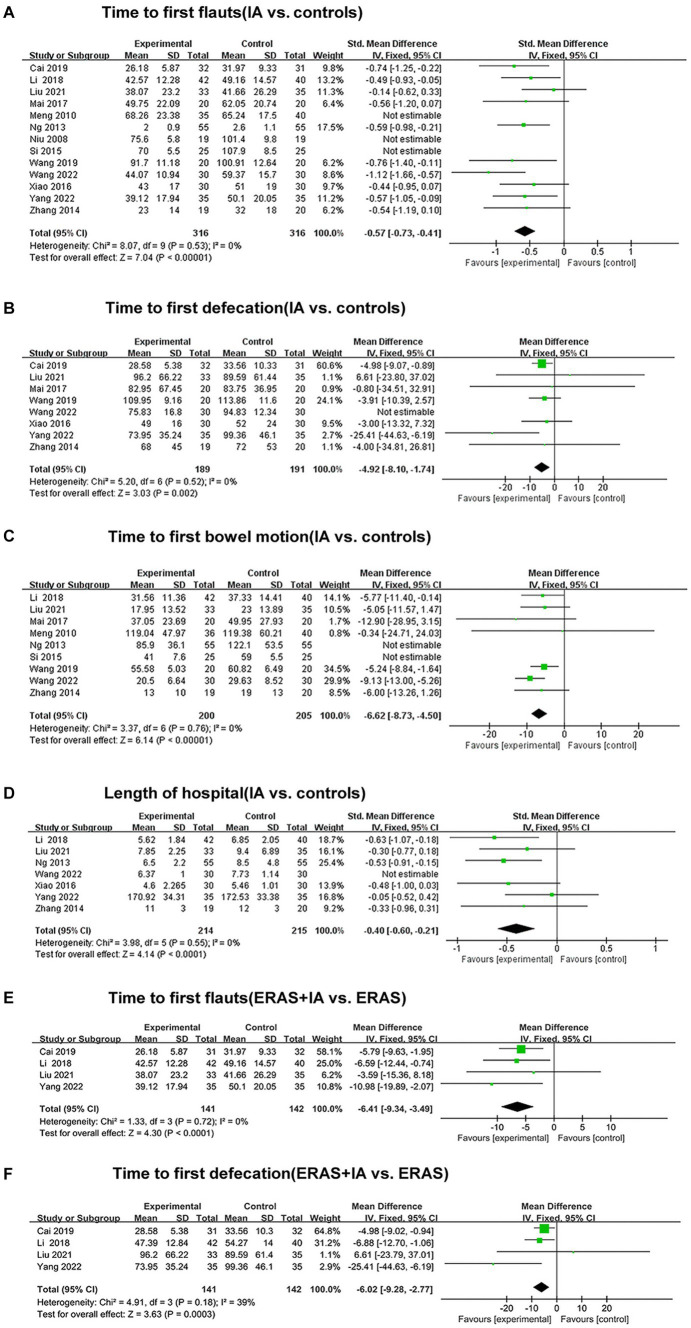
The forest plots. **(A)** Time to first flauts (IA vs. controls). **(B)** Time to first defecation (IA vs. controls). **(C)** Time to first bowel motion (IA vs. controls). **(D)** Length of hospital (IA vs. controls). **(E)** Time to first flauts (ERAS + IA vs. ERAS). **(F)** Time to first defecation (ERAS + IA vs. ERAS).

All studies reported the effect of IA vs. B/S on the time to first flauts after CRC surgery, involving 632 patients, 316 in the IA group and 316 in the B/S group. Heterogeneity was detected (*p* < 0.00001, *I^2^* = 88%) with significant heterogeneity, and sensitivity analysis was performed and three studies were excluded before heterogeneity was detected (*p* = 0.53, *I^2^* = 0%) with no significant statistical heterogeneity, low sensitivity, and good stability. The fixed-effects model combined with effect size analysis was used, and the results showed that the time to the first flauts was significantly lower in the IA group than in the B/S group [stand mean difference (SMD), −0.57; 95% CI, −0.73 to −0.41, *p* < 0.00001], as shown in Figure 3A.

#### 3.3.2. Time to the first defecation

Eight studies reported the effect of IA vs. B/S on the time to first defecation after CRC surgery: 380 patients were involved, 189 in the IA group and 191 in the B/S group. Heterogeneity was detected (*p* = 0.02, *I^2^* = 58%) with significant heterogeneity, and sensitivity analysis was performed and one study was excluded before heterogeneity was detected (*p* = 0.52, *I^2^* = 0%) with no significant statistical heterogeneity, low sensitivity, and good stability, and the fixed-effects model combined with effect size analysis was used, and the results showed that the time to the first defecation was significantly lower in the IA group than in the B/S group [mean difference (MD), −4.92 h, 95% CI −8.10 to −1.74 h, *p* = 0.002], as shown in Figure 3B.

### 3.4. Secondary outcome indicators

#### 3.4.1. Time to the first bowel motion

Nine studies reported the effect of IA vs. B/S on the first bowel motion after CRC surgery: 405 patients were involved, 200 in the IA and 205 in the B/S group. Heterogeneity was detected (*p* < 0.00001, *I*^2^ = 80%) with significant heterogeneity, and sensitivity analysis was performed and two studies were excluded before heterogeneity was detected (*p* = 0.76, *I^2^* = 0%) with no significant statistical heterogeneity, low sensitivity, and good stability, using fixed-effects model combined with effect size analysis, the results showed that the time to the first bowel motion in the IA group was significantly lower than that of the B/S group (MD, −6.62 h, 95% CI −8.73 to −4.50 h, *p* < 0.00001), as shown in Figure 3C.

#### 3.4.2. Length of hospital

Seven studies reported the effect of IA vs. B/S on the number of days in hospital: 429 patients were involved, 214 in the IA and 215 in the B/S group. Heterogeneity was detected (*p* < 0.06, *I^2^* = 50%) with significant heterogeneity, and sensitivity analysis was performed and one study were excluded before heterogeneity was detected (*p* = 0.55, *I^2^* = 0%) with no significant statistical heterogeneity, low sensitivity, and good stability, using fixed-effects model combined with effect size analysis, and the results showed that length of hospital was significantly lower in the IA group than in the B/S group (SMD, −0.40, 95% CI −0.60 to −0.21, *p* < 0.0001), as shown in Figure 3D.

### 3.5. Subgroup analysis results

Four studies reported the effect of IA combined with ERAS group on the time to the first flauts: 283 patients were involved, 141 in the IA combined with ERAS group and 142 in the ERAS group. Heterogeneity was detected (*p* = 0.72, *I^2^* = 0%), with no significant statistical heterogeneity, low sensitivity, and good stability, using the fixed-effects model combined with effect size analysis, which showed that the time to the first flauts was significantly lower in the IA combined with ERAS group than in the ERAS group (MD, −6.41 h, 95% CI −9.34 to −3.49 h, *p* < 0.0001), as shown in Figure 3E.

Four studies reported the effect of IA combined with ERAS group on the time to the first defecation: 283 patients were involved, 141 in the IA combined with ERAS group and 142 in the ERAS group. Heterogeneity was detected (*p* = 0.18, *I^2^* = 39%), with no significant statistical heterogeneity, low sensitivity and good stability, using the fixed-effects model combined with effect size analysis, which showed that the time to the first defecation was significantly lower in the IA combined with ERAS group than in the ERAS group (MD, −6.02 h, 95% CI −9.28 to −2.77 h, *p* = 0.0003), as shown in Figure 3F.

### 3.6. Publication bias

**Figure 4:**
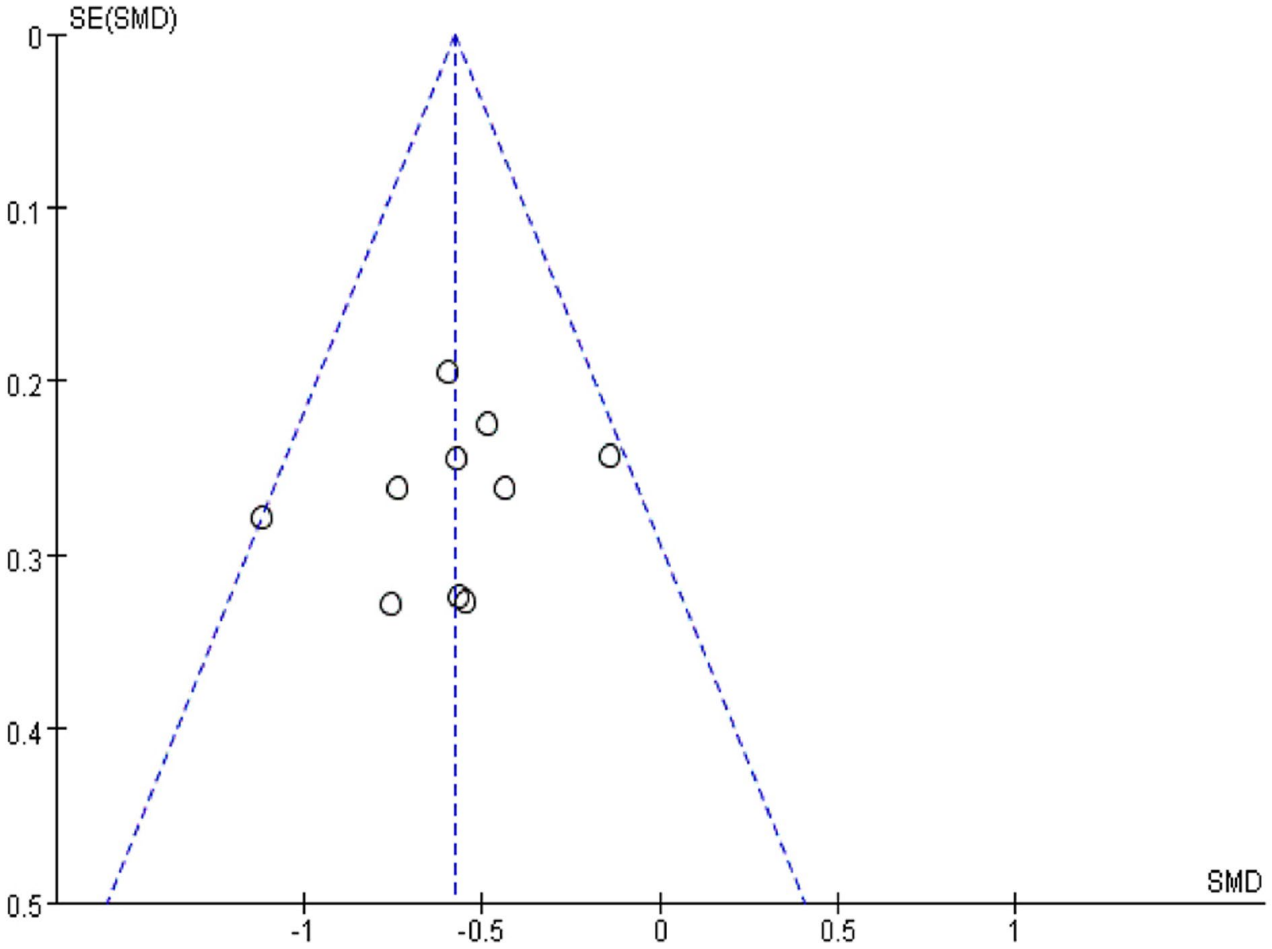
The funnel plot of comparison of the time to first flauts between IA and the B/S group.

Using Revman 5.3 software, a funnel plot was drawn for “comparing the effect of IA and B/S on the time to first flauts after CRC surgery” (Figure 4), which showed good symmetry on both sides of the central axis without significant publication bias and good reliability of the Meta-analysis results.

## 4. Discussion

Acupuncture and related therapies are guided by Traditional Chinese Medicine (TCM) and are used to prevent and treat diseases by stimulating meridians and acupuncture points, and are used in treatment guidelines for cancer pain, post-operative pain, etc. (24, 25). Currently, some studies have proposed that acupuncture has an ameliorative effect on POI after abdominal surgery such as CRC (7, 26). However, acupuncture, in its broadest sense, involves acupuncture, moxibustion, acupoint application, physical and chemical therapy, and many other means. Acupuncture interventions are mixed and vary greatly among studies due to different interventions. This study was the first meta-analysis of IA for the prevention and treatment of POI after CRC surgery. In addition, ERAS can significantly reduce the incidence of post-colorectal complications and thus has been widely used in the post-surgical period (27, 28). We also compared for the first time the effect of ERAS combined with IA with that of ERAS alone for the prevention and treatment of POI. We concluded that IA can improve POI after CRC surgery, and IA combined with ERAS can significantly reduce the time to the first flauts and defecation compared with ERAS alone, which also suggests the potential of invasive needling in combination with ERAS.

A total of 24 acupoints were involved in the 13 included studies, and the four most frequently used acupoints were as follows: ST36 (92.3%, 12/13), ST37 (50%, 6/12), ST25 (50%, 6/12), and PC6 (50%, 6/12). ST36 and ST37 are located in the lower extremities, and ST25 is located in the abdomen. All the three acupoints belong to the stomach meridian. PC6 is located in the upper extremity and belongs to the pericardium meridian. In TCM, the points mentioned above can regulate the activities of abdominal organs, and some experiments suggested that these points can promote gastric or intestinal motility and help with inflammation: Lu et al. used a vagus used a rat model in which the vagus or sympathetic nerve was removed and found that EA stimulation of PC6 could promote vagal electrical activity to increase gastric motility (29). Murakami et al. used EA stimulation on ST36 and PC6 in a rat model where the animals came down with POI after undergoing Intestinal manipulation (IM) surgery and came to the conclusion that EA stimulation improved the regularity of small intestinal slow waves, accelerated intestinal transit and gastric emptying, and inhibited TNF-α levels (30). However, EA stimulation of acupoints in the abdomen and lower extremities at the same frequency may produce different therapeutic effects. Yang set a mouse model where the animals came down with POI after undergoing sham Intestinal manipulation. In this experiment, 10 hz electrical acupuncture was given to stimulate acupuncture points ST25, ST36, and ST37, and the results suggested that both ST36 and ST37 could promote intestinal motility and reduce plasma inflammatory factors TNF-α and IL6, while ST25 had no significant effect (31). The difference in therapeutic effects may be related to the different anti-inflammatory physiological mechanisms involved in the different location of points. By identifying Prokr2 sensory neurons in the abdominal fascia and deep fascia of the hind limbs in mice, Liu et al. determined that stimulation on ST36 can be more effective than that on ST25 to activate the vagal-adrenal network, which is thought to stimulate the production of substances such as catecholamines and produce anti-inflammatory effects (32). Furthermore, in a lipopolysaccharide induced systemic inflammation mouse model, Liu et al. (33) found that EA stimulation of ST25 activated sympathetic nerves connecting the spinal cord to the spleen, producing norepinephrine. In conclusion, more distinct therapeutic effects found in different acupoints, optimal treatment frequency, and active clinical trials with relevant evidence may be of significance in improving the efficacy of acupuncture against POI.

Wang, Si′s study had significant heterogeneity in two meta-analyses and Meng, Niu’s study had significant heterogeneity in one meta-analysis, respectively. Among all studies, Meng’s study was the only one that reported no significant difference in POI improvement in the IA vs. blank group, which may be the source of heterogeneity. In addition, studies with negative results may have had publication omissions that tilted the final studies included in the analysis toward positive studies, leading to publication bias. The studies by Niu, Si, and Wang either only mentioned randomization or did not mention randomization when describing the randomization method, and their selectivity bias was evaluated as unclear and high risk. The remaining studies were specific in describing their randomization methods and all were at low risk of selective bias when evaluated. The selective bias resulting from the irregular randomization method may explain the heterogeneity of the three studies. In addition, the number of acupuncture points selected, the type of acupuncture points, the frequency and intensity of electroacupuncture, and the standardization of the ERAS protocols varied among the studies included in the analysis, which could be a source of heterogeneity in outcome indicators.

Though a subgroup analysis was performed to adjust the bias, the following limitations and shortcomings will reduce the reliability of the results. The SMD was used for time to first flatus and length of hospital stay, which leaves the final combined results without a unit of measurement, so the results for these two should be viewed with caution. In addition, all the studies are from China and there was a lack of high-quality studies reported from different countries, which may lead to selection bias. A web of science-based bibliometric study on electroacupuncture suggested that 65.1% of the electroacupuncture literature was published in China in the last decade, compared to 16.4% in the second place (34). This indicates that more countries need to be involved in acupuncture-based research. Second, there was a lack of multicenter clinical studies reported in the included studies. Third, most studies ignore allocation concealment and do not mention blinding of subjects, investigators, and assessors, which may result in selection bias, implementation bias, and measurement bias in the studies included in the analysis. Last, some of the literature does not report the basis of sample size estimation, and there is a possibility of inaccurate sample size. In conclusion, future studies need to follow the STRICTA and CONSORT statements to design and conduct trials that provide more multicenter, large-sample clinical studies with standardized outcome indicators. Further validation of the effectiveness of POI for invasive treatment of CRC surgery is still needed to provide a lower bias and higher quality evidence-based medical rationale.

## 5. Conclusion

This study suggested that IA can improve POI of CRC. The implementations of acupuncture usually start from 1 postoperative day 1 until the ileus ending, 1–2 times a day, about 30 min minutes, and 2–10 acupoints are selected (ST36 is the most important acupoint). In addition, ERAS combined with the above IA treatment was more effective than ERAS alone in preventing POI. This meta-analysis was based on five high-quality RCTs and eight low quality RCTs with blinding defects, and further validation is still needed by including more high quality studies in the future.

## Data availability statement

The original contributions presented in the study are included in the article/Supplementary material, further inquiries can be directed to the corresponding author.

## Author contributions

XZ and SS helped to conceive, design, analyze the data, conduct the study, and write the manuscript. JL helped to design the study and review the manuscript. XL and DZ designed, conducted, and reviewed the manuscript. YM and AH helped to design, conduct, and decide the final manuscript. All authors contributed to the article and approved the submitted version.

## Conflict of interest

The authors declare that the research was conducted in the absence of any commercial or financial relationships that could be construed as a potential conflict of interest.

## Publisher’s note

All claims expressed in this article are solely those of the authors and do not necessarily represent those of their affiliated organizations, or those of the publisher, the editors and the reviewers. Any product that may be evaluated in this article, or claim that may be made by its manufacturer, is not guaranteed or endorsed by the publisher.
